# Mitochondrial Bioenergetics at the Onset of Drug Resistance in Hematological Malignancies: An Overview

**DOI:** 10.3389/fonc.2020.604143

**Published:** 2020-12-21

**Authors:** Alessandro Barbato, Grazia Scandura, Fabrizio Puglisi, Daniela Cambria, Enrico La Spina, Giuseppe Alberto Palumbo, Giacomo Lazzarino, Daniele Tibullo, Francesco Di Raimondo, Cesarina Giallongo, Alessandra Romano

**Affiliations:** ^1^ Department of Clinical and Experimental Medicine, University of Catania, Catania, Italy; ^2^ Department of General Surgery and Medical-Surgical Specialties, University of Catania, Catania, Italy; ^3^ Department of Medical, Surgical Sciences and Advanced Technologies G.F. Ingrassia, University of Catania, Catania, Italy; ^4^ Saint Camillus International University of Health and Medical Sciences, Rome, Italy; ^5^ Department of Biotechnological and Biomedical Sciences, University of Catania, Catania, Italy; ^6^ Department of Surgery and Medical Specialties, University of Catania, Catania, Italy

**Keywords:** OX-PHOS, mitochondria, multiple myeloma, acute myeloid leukemia, chronic lymphatic leukemia, lymphoma

## Abstract

The combined derangements in mitochondria network, function and dynamics can affect metabolism and ATP production, redox homeostasis and apoptosis triggering, contributing to cancer development in many different complex ways. In hematological malignancies, there is a strong relationship between cellular metabolism, mitochondrial bioenergetics, interconnections with supportive microenvironment and drug resistance. Lymphoma and chronic lymphocytic leukemia cells, e.g., adapt to intrinsic oxidative stress by increasing mitochondrial biogenesis. In other hematological disorders such as myeloma, on the contrary, bioenergetics changes, associated to increased mitochondrial fitness, derive from the adaptive response to drug-induced stress. In the bone marrow niche, a reverse Warburg effect has been recently described, consisting in metabolic changes occurring in stromal cells in the attempt to metabolically support adjacent cancer cells. Moreover, a physiological dynamic, based on mitochondria transfer, between tumor cells and their supporting stromal microenvironment has been described to sustain oxidative stress associated to proteostasis maintenance in multiple myeloma and leukemia. Increased mitochondrial biogenesis of tumor cells associated to acquisition of new mitochondria transferred by mesenchymal stromal cells results in augmented ATP production through increased oxidative phosphorylation (OX-PHOS), higher drug resistance, and resurgence after treatment. Accordingly, targeting mitochondrial biogenesis, electron transfer, mitochondrial DNA replication, or mitochondrial fatty acid transport increases therapy efficacy. In this review, we summarize selected examples of the mitochondrial derangements in hematological malignancies, which provide metabolic adaptation and apoptosis resistance, also supported by the crosstalk with tumor microenvironment. This field promises a rational design to improve target-therapy including the metabolic phenotype.

## Introduction

Since the first description by Rudolf Albrecht von Kölliker in 1857, scientists have explored the essential roles of mitochondria in cell biology, as the powerhouse of the cells able to produce comparing weight to weight, thousands of times more energy per second as compared to sun production (1). Mitochondria can fuel cellular energy demands by using as substrate pyruvate, arising from glycolysis or lipolysis coupled to β-oxidation of fatty acids (FA), in the oxidative-phosphorylation (OX-PHOS) process coupled to the electron transport chain (ETC).

The combined derangements in mitochondria network function and dynamics can affect metabolism and ATP production, redox homeostasis and apoptosis triggering, contributing to cancer development in many different complex ways (2). In cancer, there is a gap in knowledge about the protein composition, structure and dynamics of lipid droplet–mitochondria structures and how bidirectional FAs exchange occur, even if the strong relationship between cellular metabolism, mitochondrial bioenergetics, and tumorigenesis development is an established emerging hallmark (2, 3).

There is a growing evidence that the metabolic reprogramming required in cancers to fuel the increased energy demand is coupled to the increased ability to evade apoptosis (2, 4). Modulating ATP availability might be an essential strategy in inducing cell resistance and sustaining cancer progression and growth (4). Recent findings have demonstrated that cancer cells take advantage of high OXPHOS, including leukemias, lymphomas, pancreatic ductal adenocarcinoma, melanoma, and endometrial carcinoma (5–16), while mitochondria can modulate their morphology regulating the intrinsic apoptotic pathway and participating in the resistance of cancer cells to apoptotic stimuli (17–27).

**Figure 1:**
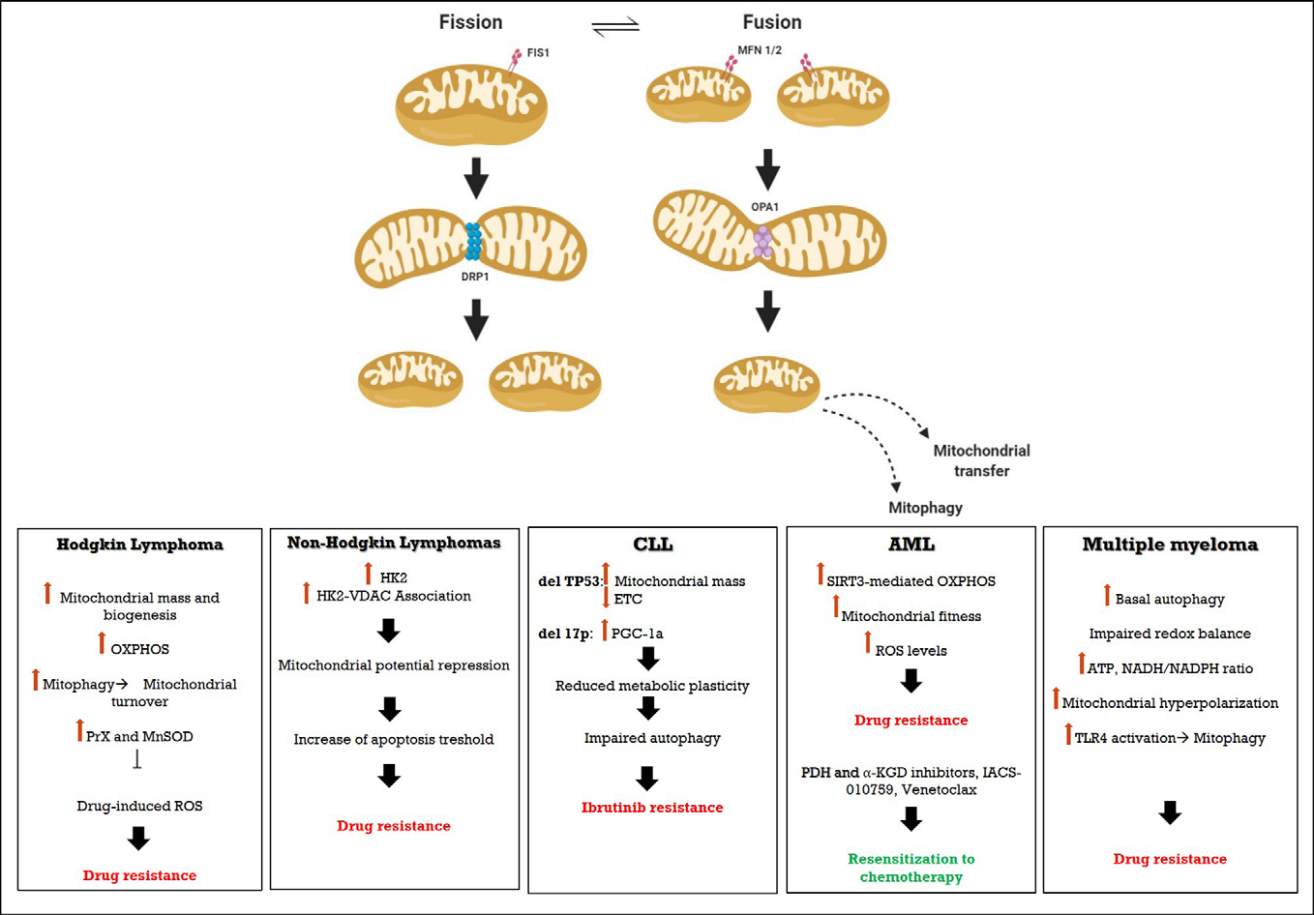
Schematic representation of Mitochondrial involvement in drug restistance in hematological malignancies.

In this review, we will summarize the most advanced body of knowledge about the mitochondrial derangements in hematological malignancies which provide metabolic adaptation and apoptosis resistance, with a particular focus on the implication of novel relevant targets to reduce the risk of recurrence (**Figure 1**).

## Mitochondria and Cancer Cell Bioenergetics

### Mitochondria and ATP Production: The Oxidative-Phosphorylation Process

The oxidative-phosphorylation (OX-PHOS) process is the electron transfer chain (ETC) driven by substrate oxidation (e.g pyruvate) coupled to the synthesis of ATP through an electrochemical transmembrane gradient. OX-PHOS is carried in the internal mitochondrial membrane (IMM) where four big multi-protein complexes, that contain flavins, iron-sulfur clusters, heme proteins, copper (Cu) structures, are organized to provide energy transformations in the ETC, namely Complex I, Complex II, Complex III Complex IV, as described in recent review for more details (20–22, 28–30), which operate together to generate water and a proton gradient, in presence of oxygen. The energy accumulated in the form of electrochemical proton gradient is used by F0-F1 ATP synthase (called Complex V also) to produce ATP from ADP and phosphate (4, 28, 31, 32).

Complex I (NADH–CoQ reductase), the largest respiratory complex, catalyzes the electron transfer from NADH to the CoQ (ubiquinone), thus to regenerate NAD^+^ levels oxidizing the NADH, which is formed both during the tricarboxylic acid (TCA) cycle and β-oxidation of fatty acids (31, 33). Complex moves four protons from the mitochondrial matrix to the intermembrane space generating a potential decrease of 360 mV (33).

Complex II (succinate dehydrogenase or succinate-ubiquinone oxidoreductase), a membrane-bound component of the Krebs cycle, permits the oxidation of the metabolite succinate to fumarate, transferring two protons to CoQ, trough the FAD/FADH2 coenzymes, thus to coupling the ETC and TCA cycle (34, 35).

Complex III (coenzyme Q: cytochrome c-oxidoreductase) catalyzes the reduction of cytochrome c, provides two protons to the CoQH2, originating from complex I and II to regenerate the CoQ, and pumps 4 protons from the mitochondrial matrix to the intermembrane space. The resulting so-called *Q cycle* allows the formation of a proton gradient across the membrane: oxidation of CoQ pumps four protons into the intermembrane space (positive side), two protons are taken up from the matrix (negative side) and two electrons are transferred from the ubiquinol to the ubiquinone, *via* two cytochrome c intermediates, to complete the cytochrome c reduction (36).

Complex IV (cytochrome c oxidase) transfers electrons from cytochrome c to oxygen to generate water and a proton gradient; its assembly and deregulation has been extensively reviewed recently (37).

Complex V consists of two main subunits: F0 is the transmembrane unit that works like a proton-driven turbine; F1 is the catalytic subunit located on the mitochondrial matrix side (38).

As a whole, OX-PHOS couples energy demands to the structural integrity of mitochondria within the cells. The membrane potential is essential for mitochondrial functions for cell survival and is well used by cancer cells to trigger molecular changes to make mitochondria network more efficient to provide energy requirements and resilience to changes in the redox status, induced by increased proliferation rate, and consequent increased nucleotide and lipid synthesis and/or drug exposure.

### Mitochondria Network and Response to Dynamic Energy Demand: Fusion, Lipid Droplets (LDs) Trafficking, and Fission

Mitochondria are small organelles devoted to homeostasis and redox status maintenance within the cell, through cellular respiration, with consequent ATP production, heat production, biosynthesis of lipids and iron-containing prosthetic group heme enzymes, and apoptosis control (39, 40). These processes happen simultaneously in different compartments, identifiable by ultrastructural hallmarks, including double lipid membranes and inner membrane folds forming “cristae”. The outer mitochondrial membrane (OMM) is a double phospholipid membrane separating the inside of the organelle from the rest of the cell while the inner mitochondrial membrane (IMM), separates the inter-membrane space from the central matrix, the site of the electron transport chain (ETC). The IMM and the OMM enclose the intermembrane space (IMS) of mitochondria.

The adaptation of mitochondrial morphology to cellular bioenergetics occurs at IMM by remodeling of mitochondrial cristae (5, 17, 41). The number of mitochondria in the cell and their distribution in the mitochondrial networks is regulated by two interconnected and highly dynamic processes: the fusion, which allows the merge of two mitochondria into one, and the fission, which allows the division of one mitochondrion in two daughter mitochondria, in response to ATP request or to release of cytochrome C, leading to cell death (18, 20–22, 42).

In metabolic active cells, mitochondrial fusion is tightly regulated by three GTP-ase enzymes of the dynamin superfamily: Mfn1 (Mitofusin-1), Mfn2 (Mitofusin-2), localized on the OMM, and OPA1 (optic atrophy 1), associated with the IMM.

In highly-energy demanding cells not all mitochondria work with the same efficiency (43, 44) and a balance between fluctuating energy demands, energy storage in LDs and utilization in mitochondria is required. Indeed, “peridroplet” mitochondria, characterized by elevated Krebs cycle activity but low FA oxidation capacity, support LDs biogenesis and protect the cell from lipotoxicity, while non-lipid droplet-bound cytoplasmic mitochondria are addressed to FA oxidation and energy production (43). The presence of two kind of mitochondria networks become relevant when starvation occurs and autophagy, which leads to bulk release of FAs, is triggered. In these scenarios, LDs provide a lipid buffering system that sequesters FAs released during the autophagic degradation of membranous organelles, reducing mitochondria lipotoxicity. In turn, FAs are removed by cytoplasmatic lipases to enable their transfer to mitochondria to provide an “on-demand” source of fatty acids for bulk ATP production (44), only in highly fused peridroplet mitochondria. If mitochondrial fusion is prevented, FAs could not be efficiently metabolized and are re-associated with LDs and fluxed. Thus, relevant to cancer metabolism, mitochondrial fusion dynamics ensures maximum oxidative metabolism and avoids FA toxicity in starved cells (44). As shown in murine brown adipose cells, lack of Mfn2 induces LDs accumulation and mitochondrial dysfunction (45). Indeed, mitochondrial fusion is required for β-oxidation of fatty acids (FA) and mitochondrial respiration (46); otherwise, LDs accumulate and FA efflux into neighboring cells (44).

The trafficking of FA between mitochondria and LDs is bidirectional, regulated by the lipid droplet-coating proteins PLIN1 and PLIN5 that, if overexpressed, promote clustering of mitochondria around LDs, by binding Mfn2 (43). In basal state, Plin1 and Plin5 participate to accumulate palmitate into triglycerides to limit its utilization by the mitochondria, by inhibiting hydrolysis and stabilizing the lipid droplet. In starvation, as consequence of protein kinase A-stimulated triggering, LD hydrolysis inhibition is lifted, and FAs are released from LDs to undergo β-oxidation in mitochondria (47, 48). This is associated to a transcriptional signaling, as well. Indeed, in response to starvation-induced lipolysis and protein kinase A-dependent translocation and enrichment of PLIN5 in the nucleus, transcriptional complexes with sirtuin 1 (SIRT1) and peroxisome proliferator-activated receptor γ co-activator 1α (PGC1α) can activate Nrf2-ARE system and promote transcription of target genes involved in mitochondrial biogenesis and oxidative metabolism (49). Finally, during starvation and autophagy activation, LDs can remove damaged lipids and proteins from mitochondria, to delay mitochondria fission and apoptosis triggering (43, 44, 49–52).

In resting cells, when metabolic requirements are reduced or when there is an insult leading to increased oxidative stress (e.g., oxidative damage, overcharge of ROS levels), mitochondrial fission is carried out by multi proteins machineries, such as the GTP-molecules Fis1 (Mitochondrial fission protein 1), which is integrated into the OMM, and Drp1 (Dynamin-related protein 1). Fis1 allows Drp1 to shuttle from the cytosol to the OMM, where it forms a ring that drives the division of the organelle, changing its function and structure, including mitochondrial outer membrane permeabilization, calcium influx and cytochrome c release. The resulting modifications occurring in both proteins and lipids damage mitochondria structures and induce the collapse of the mitochondrial membrane potential. The increased number of fragmented organelles in the mitochondrial network can then to be removed by a specialized form of autophagy, called mitophagy (13, 22, 26, 42, 53). Thus, mitophagy It plays a major role in maintaining a proper mitochondrial turnover by degrading damaged organelles and promoting a stable cellular pool of excellent working organelles (20, 27, 54), leading to ineffectiveness of drugs impairing mitochondrial function and consequently to chemotherapy resistance in cancer.

### Warburg Effect and its Relationship to Mitochondrial Metabolism

In cancer cells, the rate of glucose uptake is dramatically increased with consequent high secretion of lactate to support malignant cell proliferation. This process, known as Warburg Effect, occurs in the presence of oxygen and performing mitochondria (55). Indeed, in the recent decade it has been demonstrated that while glycolysis is drastically increased in tumor cells, mitochondria fitness continues to operate normally (PMID: 25277420). This upregulation of glycolysis is not just for ATP production, but also for synthesis of biomass and the production of NADPH to reduce ROS and oxidative stress.

A symbiotic relationship exists among tumor and cancer-associated fibroblasts (CAFs). In this “two-compartment” model, cancer cells and CAFs become metabolically coupled (reverse Warburg effect). High production of ROS in tumor cells promotes the oxidative stress in CAFs inducing their metabolic reprogramming associated to increased aerobic glycolysis and production of energy-rich fuels such as pyruvate, lactate and fatty acids, which in turn support the OX-PHOS in cancer cells (56). Conversely, cancer cells take up these energy-rich metabolites, which in turn enter in the tricarboxylic acid (TCA) pathway, sustain ATP production by OX-PHOS, and in overall increase cell fitness for cell growth and migration (57–63).

Relevant for hematological malignancies, in the bone marrow there is a physiological dynamic, inverse metabolic state, based on mitochondria transfer, between hematopoietic stem and progenitor cells and their supporting stromal microenvironment during quiescence, proliferation and differentiation of these two populations (64, 65), in response to lactate in the extracellular space (66).

This mitochondrial transfer has been described to sustain oxidative stress associated to proteostasis maintenance in multiple myeloma (67, 68) and acute myeloid leukemia, associated to drug resistance and disease recurrence (39, 69–74).

## Increased OX-PHOS in Hodgkin Lymphoma is Associated to Reverse Warburg Effect Promoting Drug Resistance

Hodgkin lymphoma (HL) is a hematopoietic neoplasm generated from B-cells, affecting secondary lymphoid tissues such as lymph nodes and spleen. Despite recent advances in the biological knowledge and targeted therapy, almost a quarter of lymphoma patients relapse, due to the complex interactions between residual neoplastic cells and the microenvironment, and the emergence of metabolic adaptive responses which mediate drug resistance and contribute to clonal selection, at different times and in the different sites of the same patient (75–79).

In classic HL (cHL) a few neoplastic cells (Hodgkin and Reed-Stenberg HRS cells) are surrounded by an inflammatory microenvironment of accessory myeloid and lymphoid cells (80–86). Compared to the normal counterpart of B cells deriving from (post-)germinal center (GC) cells, HRS have more mitochondrial mass, e.g., more TOMM20 (Transporter of the outer mitochondrial membrane 20) and mitochondrial biogenesis proteins, upregulate some OX-PHOS key proteins (11), have dismal lactate production (87), but increased expression of lactate importer MCT1 (monocarboxylate transporter 1) (11). As consequence, HRS metabolic profile is characterized by high ATP production, as consequence of high OX-PHOS (87).

Increased OX-PHOS is associated to increased basal autophagy flux and increased mitochondrial turnover *via* mitophagy, which provides to maintain high quality mitochondria and metabolic intermediates conveying drug resistance (88). Carbon skeletons can be further provided by the supportive microenvironment, since the surrounding cells in HL exhibit high-glycolytic activity, associated to increased lactate dehydrogenase activity and lactate release, with increased lactate exporter MCT4 expression (11). The induction of Warburg effect in the microenvironment and the reverse Warburg effect in HRS cells could mediate drug resistance to chemotherapy drugs that disrupt OX-PHOS function such as doxorubicin or affect the cellular redox state (11, 89).

Drugs commonly used in the first-line treatment, such as bleomycin, doxorubicine, and vinblastine, part of ABVD regimen (85, 90–92), generate large amount of reactive oxygen species, causing oxidative stress and apoptosis (93), but increasing at the same time the expression of antioxidant enzymes that could contribute to chemoresistance in HRS cells. Bur et al., assessed expression of peroxiredoxin (Prx) II, Prx III, Prx V, Prx VI, and manganese superoxide dismutase (MnSOD) in 99 cases of uniformly treated HL. Prxs I–VI participate in cellular antioxidant defense by reducing alkyl hydroperoxides and hydrogen peroxide to the corresponding alcohol and water, while MnSOD catalyzes the dismutation of superoxide to hydrogen peroxide and oxygen and is the most important antioxidant enzyme in mitochondria, where oxidative stress is most evident under physiological circumstances, owing to oxidative phosphorylation (94). Data reported by Bur *et al.*, suggest that the induction of mitochondrial located antioxidant enzymes (MnSOD and Prx III) in both HRS cells and in reactive cellular infiltrate is significantly induced in the most aggressive cases. The evidence of a low rate of complete response to ABVD treatment in patients with low Prx V expression is therefore in line with the role of oxidative stress in the mechanism of action of these drugs. More precisely, all patients with low cytoplasmic Prx V expression in RS cells achieved CR, whereas the CR rate was highly low in those with high cytoplasmic Prx V expression in RS cells (95). Thanks to their mitochondria, HRS can sustain substantial amount of oxidative stress, and, in line with this, mitochondrial Prx V expression is related to a poor response to ABVD chemotherapy.

The reverse Warburg effect in cHL could be overcome by drugs which target glycolysis in the microenvironment (e.g., arsenic or metformin) which become synergic with other agents directed against the crosstalk between neoplastic cells and the environment, like check-point inhibitors (96, 97). The high-glycolytic activity in HL microenvironment is clinically relevant, since and associated to prognostic meaning of 18-FDG-PET persistent positivity after first cycles of chemotherapy (81–83, 98).

Taken together these data reflect the high involvement of mitochondria in the resistance to the drugs commonly used for the treatment of cHL, making them a highly favorable target for therapeutic manipulation *via* biguanides and metformin (99).

## OX-PHOS Identifies Metabolic Subtypes of B-Cell Non-Hodgkin Lymphomas

Non-Hodgkin lymphoma (NHL) includes quite heterogeneous group of blood neoplasms that differ for metabolism, cell of origin, clinical course, and response to treatment (79, 100–102). The relationship between metabolic pathway differences and drug resistance has led to an increasing interest in metabolic mechanisms important for lymphoma cells surviving.

A recent *in vitro* study about nine different B cell NHL cell lines has revealed their capacity to use glucose or glutamine as source of energy to sustain high proliferative rate. The capacity to use different substrates is related to differences in metabolic pathways. Particularly, glutamine-addicted cells use mitochondrial metabolism, while glucose-addicted cells have glycolytic metabolism also in presence of oxygen (Warburg effect), while cells that can use glutamine or glucose equally have a higher metabolic plasticity that allows them to use one or another pathway depending on the substrates availability (103).

In diffuse large B cell lymphoma (DLBCL), metabolic diversity is related to different expression profiles identified as a three-consensus cluster today used for DLBCL classification: B cell receptor (BCR)/proliferation cluster (BRC-DLBCL), oxidative phosphorylation cluster (OX-PHOS DLBCL), and host response (HR) cluster (102). Indeed, BCR-DLBCLs shows a higher expression of many component of the BCR signaling (102, 104, 105) and it is linked to the downstream PI3K/AKT/mTORC1 pathway involved in regulation of pro-survival factors, glucose acquisition, and glycolysis flux activation (14, 106, 107). Conversely, OX-PHOS DLBCLs shows an increase in mitochondrial activity and mitochondrial fatty acid oxidation (14). However, lymphoma cells show an important metabolic plasticity, and acute inhibition of BCR signaling increases glutamine catabolism fuelling the TCA cycle and palmitate-induced mitochondrial oxygen consumption (14). Increased contribution of mitochondria for energy production is related to differential activity or efficiency of mitochondrial ETC complexes which are encoded by nuclear and mitochondrial independently transcribed and translated genomes, with the exception of complex II (9, 14). The high expression of components of mitochondrial translation machinery is fundamental for OX-PHOS cells maintenance, so inhibition of mitochondrial translation machinery causes ROS production responsible of cell death (9). Moreover, lymphoma cell lines are able to regulate their metabolic activity in relation of oxygen availability. Indeed, OX-PHOS DLBCL can become resistant to hypoxic stress thanks to an increasing of Hexokinase II (HK2) expression upon eIF4E1 and HIF1α regulation, related to a high involvement of glycolysis pathway (108, 109). Gu and colleagues found that rituximab resistance in cell lines of lymphoma (110, 111) was associated to the impaired glucose metabolism, due to overexpression of HK2, which interacts with the protein of the mitochondrial outer membrane VDAC (voltage-dependent anion channel), to repress the mitochondrial membrane potential and increase the mitochondrial apoptosis threshold (112). Targeting HK2 resulted in decreased mitochondrial membrane potential, ATP production, cell viability, and re-sensitization to chemotherapy agents, suggesting that overexpression of HK2 could be a novel potential therapeutic target in rituximab-refractory lymphomas (111). Analysis of expression profile of newly diagnosed DLBCL has showed that glyceraldehyde-3-phosphate dehydrogenase (GAPDH) is related to metabolic profile of lymphoma cells. Particularly, GAPDH expression is significantly correlated with the percentage of ATP generated from glycolysis, so low GAPDH level is related to oxygen consumption in OX-PHOS -DLBCL while high GAPDH level is related to lactate production in BCR-DLBCLs. Moreover, the increased activation of mTORC1 activity in OX-PHOS-DLBCL is associated with the increase in glutamine transport rate, reduction of intracellular metabolites involved in glycolysis (G6P, G3P, and lactate) and non-oxidative arm of the pentose phosphate pathway (113).

The not-uniform metabolic behavior of DLBLC has clinical implications: first, the limited role of early PET positivity during treatment, that could reflect residual glycolytic activity of neoplastic versus microenvironment cells, based on cell of origin; second, the suboptimal results of lymphoma treatment, as shown by the association between relation between gene expression related to mitochondrial energetic function and R-CHOP resistance (113); third, the metabolic rewiring associated to the residual activity of the B-cell receptor. Casola and colleagues demonstrated that the two lymphoma clusters have a different fitness. Indeed, BCR^+^ cell lines have a higher competitive fitness than BCR^-^ counterparts thanks to BCR/PI3Kδ axis activation, which induces glycogen synthase kinase-3β phosphorylation (114) and regulates the transcriptional program, under MYC control, for the expression of OX-PHOS genes, which use carbon-skeleton of glutamine to fuel TCA cycle. Indeed, the competitive advantage of BCR- clones on BCR+ clones was due to increased glutamine catabolism, that could be observed also in absence of BCR/PI3Kδ axis through a constitutive activation of RAS/MAPK pathway (115).

## Metabolic Rewiring in Chronic Lymphatic Leukemia

Chronic Lymphocytic leukemia (CLL) is due to the clonal expansion and accumulation of malignant B-cell lymphocytes in the blood stream and in homing tissues, such as bone marrow and lymphoid organs. Circulating CLL lymphocytes are quiescent and dependent on intrinsic survival factors and proliferate when they enter homing tissues, revealing a challenge for the design of therapeutic interventions that target intrinsic survival pathways (116). Circulating and homing require a plastic metabolic rewiring that allows cells to modify their metabolism and fulfill the requirements needed to sustain survival, differentiation or proliferation (117, 118). To this end, the number of mitochondria, the total mitochondrial mass, biogenesis, bioenergetics (basal, maximal, and ATP-linked respiration rates), membrane potential and ROS are increased in CLL cells compared to naïve B-lymphocytes (119). As discussed above, also CLL cells use preferentially the reverse Warburg effect, relying primarily on OX-PHOS for generating energy (120). Clinically relevant, 18-FDG-PET is not always informative to evaluate disease burden and it is indicated only when Richter’s Syndrome or transformation to another aggressive B-cell lymphoma is suspected (121).

Using NanoString technology, it was shown that CLL lymphocytes display heightened expression of mitochondrial IDH3 and citrate transporter (SLC25A1) which yield α-ketoglutarate from isocitrate and cytoplasmic export of citrate respectively (122). ZAP-70+ CLL cells exhibited significantly higher bioenergetics than B lymphocytes or ZAP-70- CLL cells and were more sensitive to the uncoupler, carbonyl cyanide-p-trifluoro-methoxyphenylhydrazone (FCCP). Univariable and multivariable linear regression analysis demonstrated that ZAP-70+ predicted increased maximal respiration. ZAP-70+ is a surrogate for B cell receptor (BCR) activation and can be targeted by ibrutinib, which is a clinically approved Bruton’s tyrosine kinase (BTK) inhibitor. Ibrutinib-treated patients exhibit decreased oxygen consumption rates (OCR) of CLL cells. similar to control B lymphocytes, suggesting that drug treatment resets the mitochondrial bioenergetics (117). Increased OX-PHOS is a resistance mechanism to BCL-2 inhibitor venetoclax, suggesting that the implementation of combinatorial therapy with metabolic modulators may overcome drug resistance (123).

## Mitochondrial Fitness Mediates Resistance to Bortezomib in Multiple Myeloma

Multiple Myeloma (MM) is a neoplastic plasma cell disorder characterized by a complex array of clinical manifestations, including hypercalcemia, renal dysfunction, anemia, and bone lesions (collectively known as CRAB symptoms), in a wide spectrum of clinical variants ranging from benign MGUS and smoldering/indolent MM, to more aggressive, disseminated forms of MM and plasma cell leukemia (124). There is no a unique driver genetic event in MM onset, but a complex variety of chromosomal and genomic rearrangements (125), occurring at different timepoints in response to external driving forces (e.g., exposure to microbes, chronic antigen stimulation, oxidative stress). Among the most frequent mutated genes, FAM46C has been involved in both mitochondrial and bioenergetics dysfunction associated to drug resistance (126), proteostasis (127), and disease onset.

Physiologically, in the plasma cell (PCs) ontogeny, immunoglobulin synthesis and survival of competitive clones relies on preserved cell bioenergetics. In response to increased poli-ubiquitinated proteins requiring autophagy triggering (128), long-lived PCs robustly engage pyruvate-dependent respiration and rely on OX-PHOS whereas their short-lived counterparts could not (129). Thus, the transition from plasmablast to short-lived and long-lived PCs is associated to increased autophagy fluxes to allow removal of damaged mitochondria and lipid droplets accumulation to maintain protein and lipid homeostasis (44). Several groups have recently showed that integrity of mitochondrial function relies on p62 to limit oxidative stress, and conversely, lack of p62 is associated with inhibited complex I mitochondrial respiration. As consequence, reduced efficiency of electron transport chain (ETC) is associated to metabolic derangement inducing pentose phosphate pathway and increased cytosolic reduced glutathione (GSH) levels. Conversely, complex I inhibition resulted in lower mitochondrial membrane potential and higher cytosolic ROS production. Pharmacological activation of transcription factor Nrf2 increased mitochondrial NADH levels and restored mitochondrial membrane potential in p62-deficient cells (130).

Relying on the detoxifying mitochondrial function is consequence of other two aberrant metabolic changes occurring in MM: the oxidative stress, consequence of the aberrant protein synthesis of incomplete immunoglobulins with defective glycosylation (131), and the lack of glutamine synthetase which confers increased ammonium production and requires nitrates detoxification (132).

In relapsed and refractory patients MM PCs overexpress mitochondrial biogenesis signatures regulated by the cellular iron content (133). The consequent loss of integrity of redox balance, with the nuclear compartmentalization of heme-oxygenase 1 is associated to drug resistance and genomic instability (134). MM can intake iron to increase their scavenger antioxidant-related genes and mitochondrial mass. Iron trafficking, by modifying energetic metabolism of cancer cells and impairing inflammatory status of macrophages in the microenvironment, is a critical regulator to reshape the MM tumor niche (135). However, to make the picture more complex, MM cell lines are characterized by distinct ferritin levels, which directly correlate with bortezomib resistance and pre-treatment with ferric ammonium citrate (FAC) decreased bortezomib sensitivity *in vitro* (135).

Bioenergetics changes, associated to increased mitochondrial biomass and function, can be elicited as part of the adaptive response to treatment (135–138). In vitro, human MM cell lines resistant to bortezomib or dexamethasone have higher concentrations of ATP, NADH/NADPH ratio, associated to hyperpolarization of mitochondrial membrane leading to impaired drug response (137, 139). In vitro, pre-treament with the OX-PHOS inhibitor tigecycline can increase bortezomib sensitivity (Alejandra Ortiz-Ruiz, ASH 2019, poster 4408) (140). Similarly, inhibition of PGC-1α (SR18292), relevant for OX-PHOS, significantly impaired the proliferation and survival of MM cells due to the energy exhaustion and oxidative damage (141). These and other preclinical studies confirm OX-PHOS as possible targets for sensitization to chemotherapy treatment in MM (142), including the efficacy of Venetoclax (BCL-2 inhibitor) that could be used independently from the genetic lesion (143). Further steps could include a metabolic classification of MM subtypes based on mitochondria number and OX-PHOS quantification (143).

Our group has recently disclosed that TLR4 acts as a mitochondria protective factor against bortezomib-induced mitochondria damage and apoptosis (144). Targeting TLR4 signaling in bortezomib resistant cells damages mitochondrial fitness and increases mitophagy leading to apoptosis. As TLR4 pathway is also activated in MM mesenchymal stromal cells (MSCs) driving their commitment toward a pro-inflammatory and pro-tumor behavior (145), TLR4 inhibition could be an adjuvant therapy to interrupt the self-reinforcing stromal changes in MM microenvironment. Taken together, changes in microenvironment composition (146) and REDOX status can affect sensitivity to novel agents (147) and should be taken in account in designing novel combinations.

## Mitochondrial Metabolism Dependency in Acute Myeloid Leukemia and Novel Therapeutic Targets

Acute myeloid leukemia (AML) is a heterogeneous disease characterized by a blockade in differentiation of hematopoietic stem cells with a clonal proliferation of myeloid blast in BM and peripheral blood. Due to the high relapse rate and poor clinical outcome, overcoming chemoresistance remains the most important goal in AML patients. Changes in cell metabolism and metabolic adaptation are a hallmark of many cancers, including AML, supporting tumor initiation, growth, and response to therapeutics. The discovery of enzymes deficiency and mutations in key metabolic enzymes has highlighted the importance of metabolism in cancer biology and how these changes might constitute a weakness for cancer treatment.

A study carried out by Chen et al., reported that some metabolites such as pyruvate and lactate were specifically enriched in the serum of patients at diagnosis compared to healthy controls and demonstrated prognostic value in cytogenetically normal AML (CN-AML) patients as it could predict poor survival for these patients (148). Interestingly, deletions of the two glycolytic enzymes PKM2 and LDHA, which catalyze the production of cytosolic pyruvate and lactate, respectively, inhibit leukemia initiation *in vivo* in AML mice models.

It has also been reported that a wide percentage of AML patients are deficient in arginosuccinate synthetase-1 (ASS1), an enzyme that allows the conversion of citrulline and aspartate into argininosuccinate (149). The loss of ASS1 has also been found in other tumor types where it is required to support cell proliferation and nucleotide synthesis by sustaining the intracellular aspartate level (150). A decrease in ASS1 can also lead to a dependence on arginine, which has been explored as a potential vulnerability in different cancer types, including AML (151).

Recent advances in cancer genetics have found mutations in the isocitrate dehydrogenase 1 (*IDH1)* and 2 (*IDH2) genes* occur frequently in a variety of human cancers, including AML. Wild-type IDH1 and IDH2 are important metabolic enzymes catalyzing the oxidative decarboxylation of isocitrate to generate α-ketoglutarate (αKG) and CO2. IDH1 represents the peroxisomes and cytosol isoform, while IDH2 is localized in mitochondria. The common function of IDH1/2 active-site mutations is a new enzyme activity that catalyzes the conversion of αKG to D-2-hydroxyglutarate (D2HG). Under physiological conditions, cellular D2HG accumulation is limited due to the actions of the endogenous D2HG dehydrogenase (D2HGDH), which catalyzes the reverse reaction from D2HG to αKG.

D2HG has been demonstrated to inhibit αKG-dependent dioxygenases that are involved in the regulation of epigenetics and differentiation and is thought to induce epigenetic dysfunction inhibiting normal cellular differentiation. Specifically, elevated D2HG levels competitively inhibit αKG-dependent lysine demethylases, resulting in elevated levels of histone methylation in a variety of cell line models (152, 153). Consequently, inhibition of cellular differentiation by D2HG is thought to promote the pathological self-renewal of stem-like progenitor cells, which may create a cellular state prone to malignant transformation.

Evidence from AML patients and preclinical models strongly suggests that *IDH1* and *IDH2* mutations are oncogenic drivers of AML and myelodysplastic syndrome and that targeting IDH mutant neomorphic activity in this context may provide therapeutic benefit by promoting the differentiation of malignant myeloid cells. Research attempts have been made to identify small molecule inhibitors of mutant IDH enzymes and to develop these molecules as drugs for anti-cancer therapy (153).

In addition to targeting metabolic enzymes, targeting OX-PHOS turned out to be a promising strategy to improve the treatment outcomes of AML. Indeed, leukemic cells have higher copy number of mitochondrial DNA, more mitochondria and increased oxygen consumption in comparison to normal hematopoietic stem cells, without a concomitant increase in respiratory chain complex activity, which confers increased susceptibility to oxidative stress (154). Acquisition of chemoresistance is associated to a shift toward a high OX-PHOS status characterized by increased mitochondrial fitness and high levels of ROS (155). Mechanistically, this can be due to increased SIRT3 expression, which significantly decreased nicotinamide adenine dinucleotide phosphate (NADP)/reduced NADP ratio and increased reduced glutathione/oxidized glutathione ratio, associated to OX-PHOS induction (156).

For those cancers, like AML, that rely on OX-PHOS, its inhibition could represent an effective therapeutic strategy. In solid cancers OH-PHOS inhibitors, including biguanides and metformin, are currently under investigation in several trials, designed to evaluate the combination of metformin with chemotherapy, as recently reviewed (157, 158). Several drugs, including biguanides, metformin, atovaquone, and arsenic trioxide, are used at clinical level for non-oncologic indications, but growing evidences indicate their potential use as OX-PHOS inhibitors (8). The detrimental effect of metformin is emerging in AML, alone (159) or in combination with cytarabine (160), and Venetoclax induced cell-cycle arrest leading to clinical trials.

CPI-613, designated as orphan drug for the treatment of peripheral T-cell lymphoma, is a lipoate analog that blocks pyruvate dehydrogenase (PDH) and α-ketoglutarate dehydrogenase (KGDH), induces collapse of mitochondrial function associated to large, tumor-specific production of mitochondrial ROS (161). Based on encouraging results in phase I clinical studies (162, 163), a phase III randomized study is ongoing in the setting of refractory/relapsing AML to compare the efficacy of standard chemotherapy supplemented or not with CPI-613. Similarly, phase I trials disclosed promising results using IACS-010759, is an ECT inhibitor, acting against complex I in AML and NHL (164).

Taken together, ongoing trials in AML and other hematological malignancies show that targeting OX-PHOS is a promising strategy to induce a metabolic rewiring leading to chemo-sensitization.

## Conclusions

Mitochondria play many important roles in cell functions and homeostasis, including the production of ATP, the release of death-promoting factors upon apoptotic stimuli and a variety of metabolic pathways. Contrary to conventional wisdom, functional mitochondria are essential for cancer cells. Although mutations in mitochondrial genes are common in cancer cells, they do not inactivate mitochondrial energy metabolism but rather alter the mitochondrial bioenergetic and biosynthetic state. These alterations activate out-of-context programs that are important in the onset and the development of malignancies. However, different cancer cell types undergo different bioenergetic alterations, some to more glycolytic and others to more oxidative, depending in part on the developmental state of the cell undergoing neoplastic transformation (165).

In most hematological malignancies, cancer cells show greater basal mitochondrial activity compared to healthy counterparts leading to higher levels of oxidative stress. ROS, adaptation to ROS, and mitochondrial biogenesis appear to form a self-amplifying feedback loop to sustain recurrence, as shown in CLL (119). However, in other settings, like MM, bioenergetics of tumor cells can change, as consequence of increased mitochondrial biomass and function, as part of the adaptive response to drug-induced stress (139).

In particular, interactions between cancer cells and surrounding microenvironment highly affect the growth, metabolism, metastasis and progression of cancer. The so-called reverse Warburg effect has been proposed to reconsider bioenergetics of cancer cells and stromal cells become metabolically coupled (56, 58, 59). In a vicious circle, neoplastic cells induce oxidative stress in neighboring microenvironment to undergo aerobic glycolysis and generate high level of energy-rich fuels (such as pyruvate, ketone bodies, fatty acids, and lactate) that fuel mitochondrial OX-PHOS in cancer cells and are utilized for efficient ATP production (56). In addition, microenvironment can contribute through horizontal mitochondrial transfer, when neoplastic cells become incapable of aerobic respiration due to defective or deleted mtDNA (166), Taking up functional mitochondria derived from the microenvironment can increase mitochondrial mass to improve metabolic fitness of neoplastic cells and conferring drug resistance.

It is probably that in next years “*mitochondrial medicine*” will play an active role to design effective therapeutic strategies to target the interplay between microenvironment and neoplastic cells to tailor the metabolic phenotype and not only the genomic aberrancies, with novel targets for selective anti-cancer therapy.

## Author Contributions

AB, GS, CG, and AR designed the paper and wrote the manuscript. FP and FR reviewed literature about myeloma. DC and ES reviewed literature about lymphoma. GP reviewed literature about AML. GL and DT reviewed literature about OX-PHOS targeting. All authors contributed to the article and approved the submitted version.

## Conflict of Interest

The authors declare that the research was conducted in the absence of any commercial or financial relationships that could be construed as a potential conflict of interest.

The handling editor declared a past co-authorship with one of the authors FR.
